# Role of Nitric Oxide and Hydrogen Sulfide in the Vasodilator Effect of Ursolic Acid and Uvaol from Black Cherry *Prunus serotina* Fruits

**DOI:** 10.3390/molecules21010078

**Published:** 2016-01-12

**Authors:** Francisco J. Luna-Vázquez, César Ibarra-Alvarado, Alejandra Rojas-Molina, Antonio Romo-Mancillas, Fabián H. López-Vallejo, Mariana Solís-Gutiérrez, Juana I. Rojas-Molina, Fausto Rivero-Cruz

**Affiliations:** 1 Laboratorio de Investigación Química y Farmacológica de Productos Naturales, Facultad de Química, Universidad Autónoma de Querétaro, Centro Universitario, Querétaro 76010, Mexico; fjlunavz@yahoo.com.mx (F.J.L.-V.); rojasa@uaq.mx (A.R.-M.); romtono@comunidad.unam.mx (A.R.-M.); fabianhlv@gmail.com (F.H.L.-V.); mariasolisgutierrez@gmail.com (M.S.-G.); jirojas@gmail.com (J.I.R.-M.); 2 Departamento de Farmacia, Facultad de Química, Universidad Nacional Autónoma de México, Ciudad Universitaria s/n, México D.F. 04510, Mexico; joserc@unam.mx

**Keywords:** hydrogen sulfide, in silico study, nitric oxide, *Prunus serotina*, ursolic acid, uvaol, vasodilation

## Abstract

The present research aimed to isolate the non-polar secondary metabolites that produce the vasodilator effects induced by the dichloromethane extract of *Prunus serotina* (*P. serotina*) fruits and to determine whether the NO/cGMP and the H_2_S/K_ATP_ channel pathways are involved in their mechanism of action. A bioactivity-directed fractionation of the dichloromethane extract of *P. serotina* fruits led to the isolation of ursolic acid and uvaol as the main non-polar vasodilator compounds. These compounds showed significant relaxant effect on rat aortic rings in an endothelium- and concentration-dependent manner, which was inhibited by NG-nitro-l-arginine methyl ester (l-NAME), dl-propargylglycine (PAG) and glibenclamide (Gli). Additionally, both triterpenes increased NO and H_2_S production in aortic tissue. Molecular docking studies showed that ursolic acid and uvaol are able to bind to endothelial NOS and CSE with high affinity for residues that form the oligomeric interface of both enzymes. These results suggest that the vasodilator effect produced by ursolic acid and uvaol contained in *P. serotina* fruits, involves activation of the NO/cGMP and H_2_S/K_ATP_ channel pathways, possibly through direct activation of NOS and CSE.

## 1. Introduction

Epidemiological studies over the last decade indicate that cardiovascular diseases are a major health problem worldwide, accounting for 30% of deaths annually [1,2,3]. In the case of Mexico, these diseases are the leading cause of overall mortality [4,5,6]. Hypertension stands out among these ailments, with a growing incidence due to the increase of risk factors in modern society [7]. Currently, there are in the market over 200 drugs to treat hypertension, but less than a third of clinical cases are treated successfully because these drugs have low effectiveness and involve various side effects [8].

Numerous evidences suggest that alterations in the function of the vascular endothelium and a decrease in the availability of endothelial relaxing factors contribute to the pathogenesis and clinical expression of hypertension, among other cardiovascular diseases [9,10,11]. Thus, there is an ongoing search for new pharmacological strategies, whose targets of action activate the signaling pathways of the main vasodilator endothelial factors [12,13], including gasotransmitters, such as nitric oxide (NO) and hydrogen sulfide (H_2_S) [14,15,16,17,18,19,20,21,22,23].

A documentary and ethno-medical study carried out by our research group indicated that among the plant species that are used in Mexico to treat cardiovascular disorders, one of the most well-known is *Prunus serotina* Ehrh (*Rosaceae*) (*P. serotina*) [24]. Therefore, our group carried out chemical and pharmacological studies of the leaves and fruits of this plant (Black cherry). A bio-directed chemical study of the methanol extract of the leaves led to the identification of hyperoside, prunin and ursolic acid as compounds with vascular smooth muscle relaxant activity [25]. We also determined that the aqueous and the dichloromethane extracts of black cherry fruits induce relaxation of vascular smooth muscle [26], which indicated that these fruits contain polar and non-polar metabolites with vasodilator effect, which may be useful for the prevention and/or treatment of cardiovascular diseases.

With respect to the characterization of polar metabolites, we found that the fruits of *P. serotina* have a high content of phenolic compounds such as chlorogenic acid, gallic acid, caffeic acid, catechin, epicatechin and quercetin and kaempferol glycosides, which are directly related to the high antioxidant capacity and significant vasodilator effect of the aqueous extract of the fruit [27].

In this context, the present research aimed to isolate the non-polar secondary metabolites that produce the vasodilator effects induced by the dichloromethane extract of *P. serotina* fruits and to determine whether the NO/cGMP and the H_2_S/K_ATP_ channel pathways are involved in their mechanism of action.

## 2. Results

### 2.1. Bio-Directed Chemical Study of the Dichloromethane Extract Obtained from the Fruits of P. serotina

**Figure 1:**
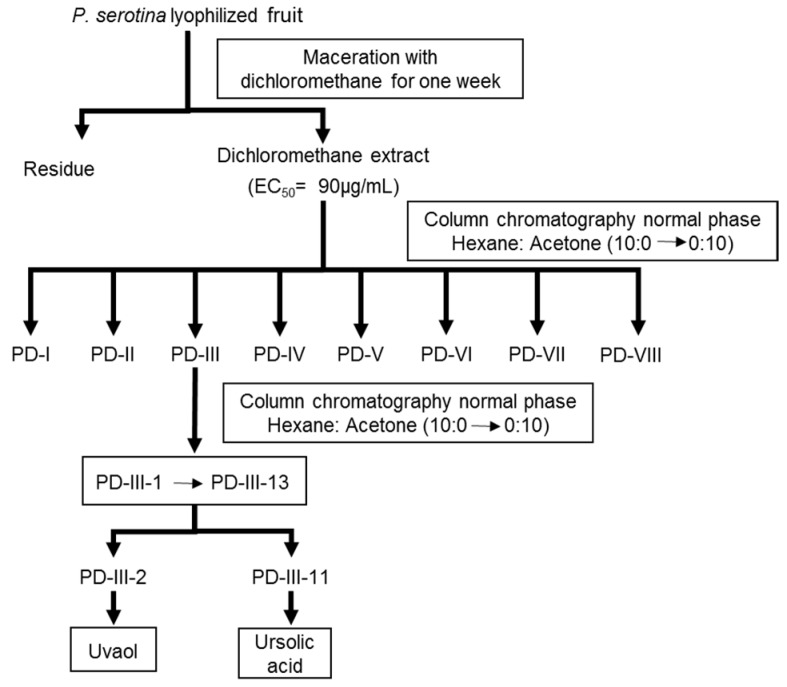
Diagram for extraction and bio-guided fractionation of the dichloromethane extract of *Prunus serotina* fruits.

The dichloromethane extract prepared from *P. serotina* fruits was fractionated by column chromatography (Figure 1), and the vasodilator activity was monitored through the isolated rat aorta assay. This assay showed that the relaxant activity on vascular smooth muscle was concentrated in the primary fraction PD-III.

**Figure 2:**
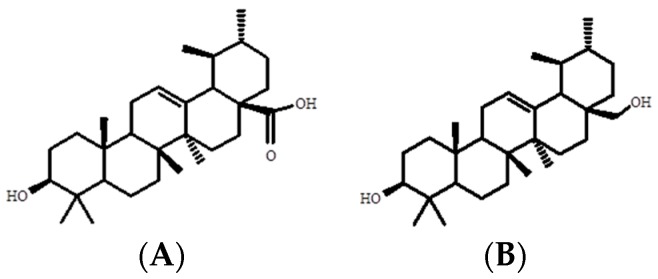
Chemical structures of ursolic acid (**A**) and uvaol (**B**).

Chromatographic analysis of fraction PD-III led to the isolation of a white compound with a melting point of 216–218 °C (fraction PD-III-2) and a white compound with a melting point of 259–260 °C (fraction PD-III-11). These compounds were identified as uvaol and ursolic acid, respectively (Figure 2), by comparing their spectroscopic data (^1^H-NMR and ^13^C-NMR) with those previously described in the literature (Table 1 and Table 2).

**Table 1:** Spectroscopic constants of ursolic acid.

^1^H-NMR (400 MHz, Acetone) δ (ppm)	δ_H_ 5.22 (1H, m, H-12), 3.15 (1H, m, H-3), 2.25 (1H, d, H-18), 1.13 (3H, s, H-23), 0.98 (3H, s, H-27), 0.96 (3H, s, H-26), 0.95 (3H, s, H-24), 0.89 (3H, d, H-29), 0.84 (3H, d, H-30), 0.78 (3H, s, H-25)
^13^C-NMR (400 MHz, Acetone) δ (ppm)	δ_C_ 177.6 (C-28), 138.4 (C-13), 125.3 (C-12), 77.7 (C-3), 55.3 (C-5), 52.9 (C-18), 47.5 (C-9), 47.3 (C-17), 41.9 (C-14), 39.5 (C-8), 38.9 (C-19), 38.9 (C-4), 38.6 (C-1), 36.8 (C-22), 36.7 (C-10), 33.0 (C-7), 30.4 (C-21), 27.9 (C-15), 27.8 (C-2), 24.1 (C-16), 23.1 (C-27), 23.0 (C-11), 20.5 (C-30), 18.2 (C-6), 16.8 (C-26), 16.6 (C-24), 15.4 (C-29), 15.0 (C-25)

**Table 2:** Spectroscopic constants of uvaol.

^1^H-NMR (400 MHz, DMSO-*d*_6_) δ (ppm)	δ_H_ 5.13 (1H, t, H-12), 3.80 (1H, d, H-20a), 3.38 (1H, d, H-28b), 3.20 (1H, d, H-3), 1.05 (3H, s, CH_3_), 0.98 (3H, s, CH_3_), 0.97 (3H, s, CH_3_), 0.96 (3H, s, CH_3_), 0.93 (3H, d, CH_3_), 0.83 (3H, d, CH_3_), 0.80 (3H, s, CH_3_).
^13^C-NMR (100 MHz, DMSO-*d*_6_) δ (ppm)	δ_C_ 177.8 (C-28),138.7 (C-13), 125.1 (C-12), 77.1 (C-3), 58.7 (C-18), 52.3 (C-5), 41.5 (C-14).17.9 (C-29), 15.9 (C-23).

### 2.2. Elucidation of the Mechanism of Action of Ursolic Acid and Uvaol

Ursolic acid (EC_50_ = 21.5 ± 3.5 µg/mL; E_max_ = 97.7% ± 3.9%) and uvaol (EC_50_ = 19.3 ± 2.5 µg/mL; E_max_ = 93.4% ± 5.1%) caused a significant concentration-dependent relaxation of the aorta. ACh (EC_50_ = 8.7 ± 0.8 µg/mL; E_max_ = 69.5% ± 5.7%), used as positive control, was more potent than ursolic acid and uvaol, but showed lower efficacy than both triterpenes.

**Figure 3:**
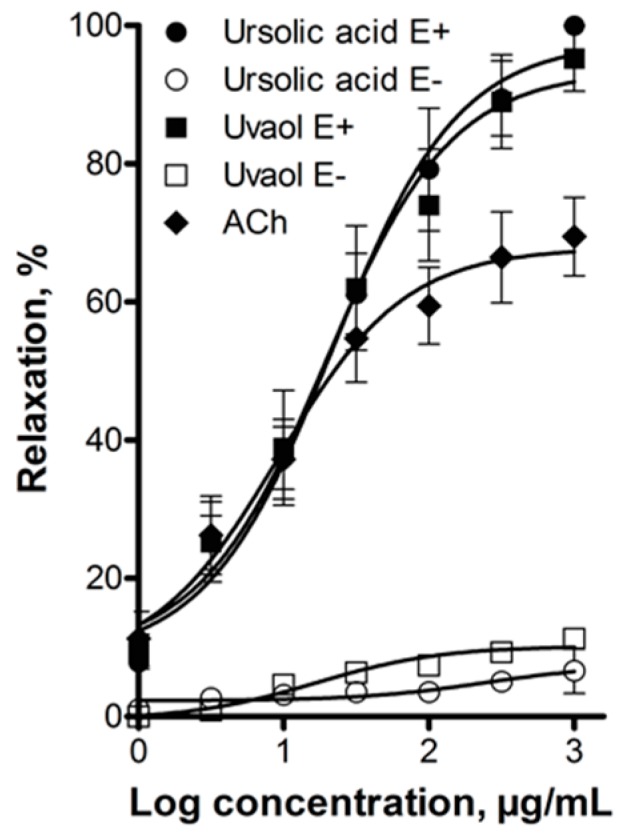
Concentration-response curves of the vasodilator effect of ursolic acid and uvaol in the presence (E+) and absence (E−) of endothelium. Acetylcholine (ACh) was used as positive control. Analysis by repeated-measures two-way ANOVA was made between the curves of each triterpene in the presence and absence of endothelium (*p* < 0.001).

Ursolic acid and uvaol relaxed the rat aorta by a mechanism that depends on the presence of the vascular endothelium, since endothelium removal reduced almost completely the vasodilator effect of these compounds (Figure 3).

**Figure 4:**
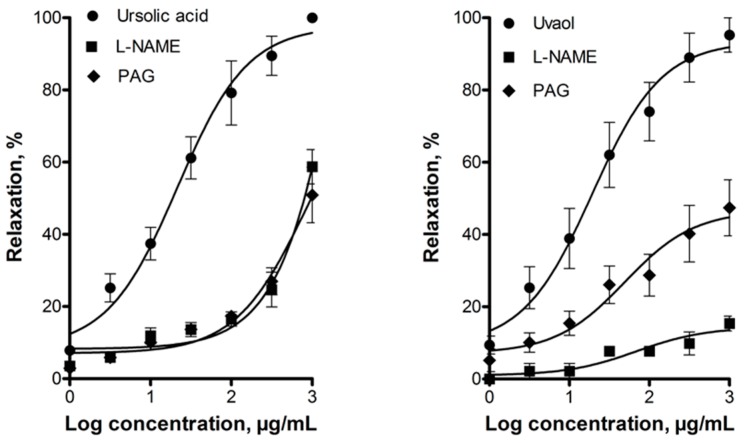
Vasodilatory effect of ursolic acid and uvaol in the absence (control) and presence of inhibitors of enzymes that produce gasotransmitters: NG-nitro-l-arginine methyl ester (l-NAME; 100 μM) and dl-propargylglycine (PAG; 10 mM). Analysis by repeated-measures two-way ANOVA was made between the curves of each triterpene in the presence of l-NAME or PAG (*p* < 0.001).

The vasorelaxant effect of these triterpenes was significantly reduced by inhibiting eNOS with NG-nitro-l-arginine methyl ester (l-NAME; 100 μM) or CSE with dl-propargylglycine (PAG; 10 mM), which indicated that the NO/cGMP and the H_2_S/K_ATP_ channel pathways are involved in this effect (Figure 4).

**Figure 5:**
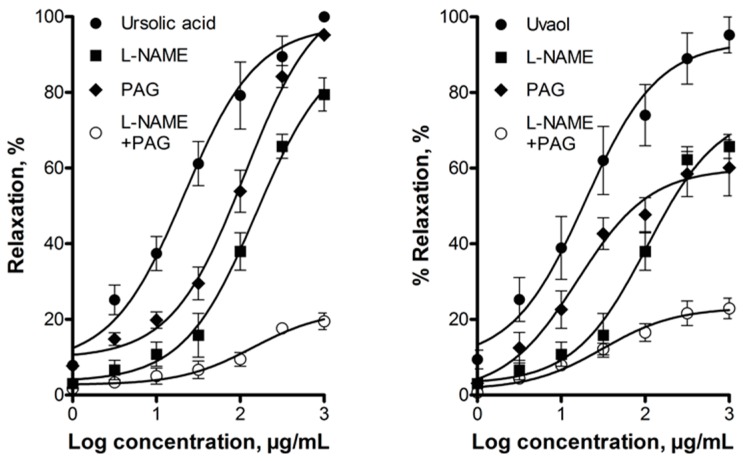
Vasodilatory effect of ursolic acid and uvaol in the absence (control) and presence of inhibitors of enzymes that produce gasotransmitters: l-NAME (1 μM), PAG (1 mM), and l-NAME (1 μM) + PAG (1 mM). Analysis by repeated-measures two-way ANOVA was made between the curves of each triterpene in the presence of l-NAME, PAG or l-NAME + PAG (*p* < 0.001).

In order to evaluate the interaction between the NO/cGMP and the H_2_S/K_ATP_ pathways in the vasorelaxant mechanism of action of ursolic acid and uvaol, submaximal concentrations of l-NAME (1 µM) and PAG (1 mM) and a mix of both inhibitors were evaluated. The simultaneous inhibition of both enzymes, eNOS and CSE, with submaximal concentrations of their respective inhibitors inhibited almost completely the vasorelaxant effect of the two triterpenes tested (Figure 5).

**Figure 6:**
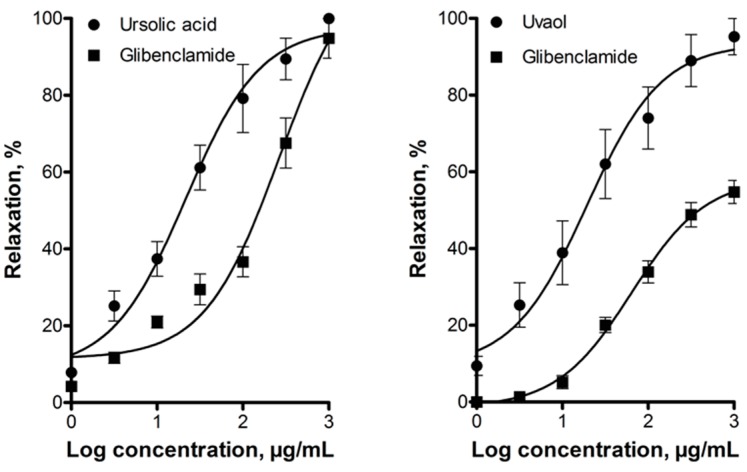
Vasodilatory effect of ursolic acid and uvaol in the absence (control) and presence of the potassium channel inhibitor glibenclamide (1 µM). Analysis by repeated-measures two-way ANOVA was made between the curves of each triterpene in the presence of glibenclamide (*p* < 0.001).

To assess whether activation of ATP-sensitive K^+^ channels was involved in the vasodilatory effect of these triterpenes, the effect of glibenclamide (a specific blocker of the K_ATP_ channels) was evaluated. This blocker significantly shifted to the right the concentration–response curves of the vasodilator effect of ursolic acid and uvaol (Figure 6), indicating that these channels are involved in this effect.

**Figure 7:**
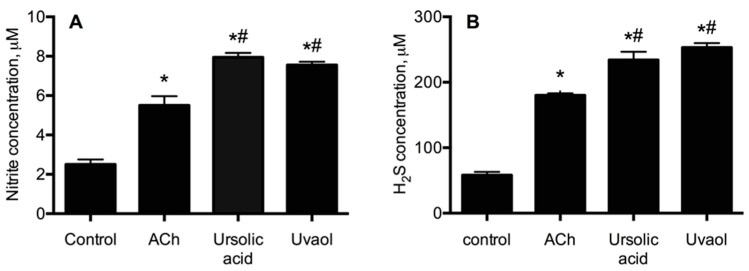
Total nitrite (**A**) and hydrogen sulfide (**B**) concentration induced by stimulation of aortic tissue with ursolic acid and uvaol. Statistical analysis was made using a one-way ANOVA, followed by a Tukey’s test (* *p* < 0.0001 *vs.* control; # *p* < 0.05 *vs.* ACh).

To confirm participation of both NO/cGMP and H_2_S/K_ATP_ channel pathways in the vasorelaxant effect of ursolic acid and uvaol, NO and H_2_S levels were quantified in the aortic tissues. As expected, the nitrite concentration increased, when aortic tissue was incubated with ursolic acid ([NO_2_^−^] = 7.95 ± 0.22 μM) and uvaol ([NO_2_^−^] = 7.55 ± 0.17 μM); both triterpenes induced a higher nitrite concentration than that of ACh ([NO_2_^−^] = 5.5 ± 0.47 μM). Similarly, quantification of H_2_S showed that stimulation of aortic tissue with ursolic acid ([H_2_S] = 234 ± 12.7 µM) and uvaol ([H_2_S] = 253 ± 6.8 µM) increased four times the H_2_S concentration with respect to the control. In the presence of ACh, H_2_S levels were only three times higher than those of the control (Figure 7).

**Figure 8:**
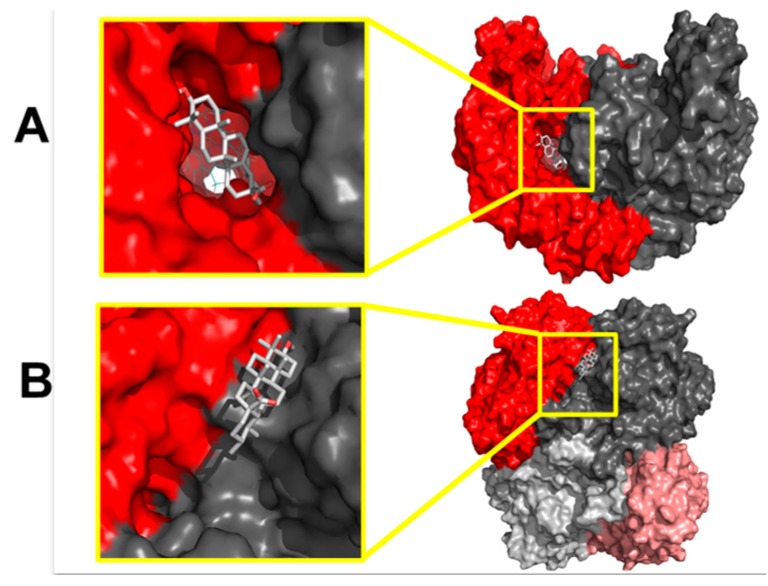
Molecular surface showing the oligomeric interfaces (color-code by chain) and the favored binding sites of triterpenes (Ursolic acid): (**A**) eNOS and (**B**) CSE. The homodimer interface is located along the region where the two monomers, labeled in different colors, connect.

Docking studies showed that ursolic acid and uvaol could bind to residues forming the oligomeric interfaces of eNOS and CSE, corresponding to the highest affinity binding sites of both enzymes. Additionally, both compounds displayed low affinity for the catalytic sites of the enzymes; however, they bind to a site close to the catalytic center, which could have an effect on the synthesis of NO and H_2_S (Figure 8).

**Figure 9:**
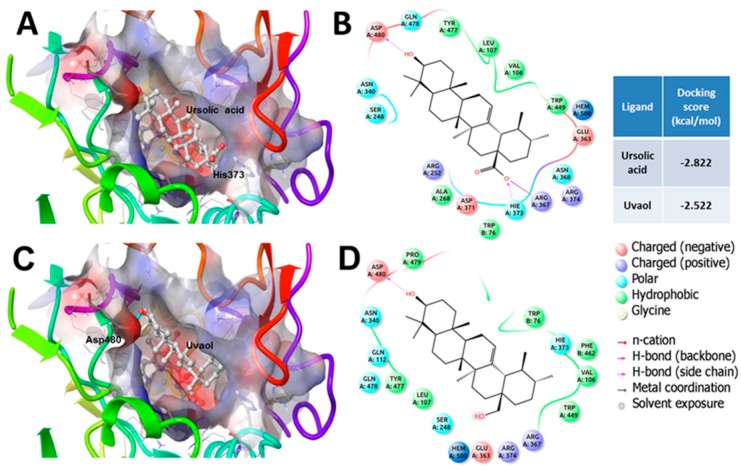
Binding mode of ursolic acid and uvaol into the eNOS binding site: (**A**) short interactions (4.5 Å) of ursolic acid into the C1 and C2 pockets; (**B**) 2D ligand interaction diagram of ursolic acid; (**C**) Short interactions (4.5 Å) of uvaol into the C1 and C2 pockets; and (**D**) 2D ligand interaction diagram of uvaol.

In eNOS, the C1 and C2 pockets where the triterpenes bind are away from the catalytic site and constitute the substrate access channel. These compounds are mostly stabilized by short contacts with residues of C1 and C2 sites and by hydrogen bonds with Asp480 and His373, as well as an electrostatic interaction with Arg367 (Figure 9).

**Figure 10:**
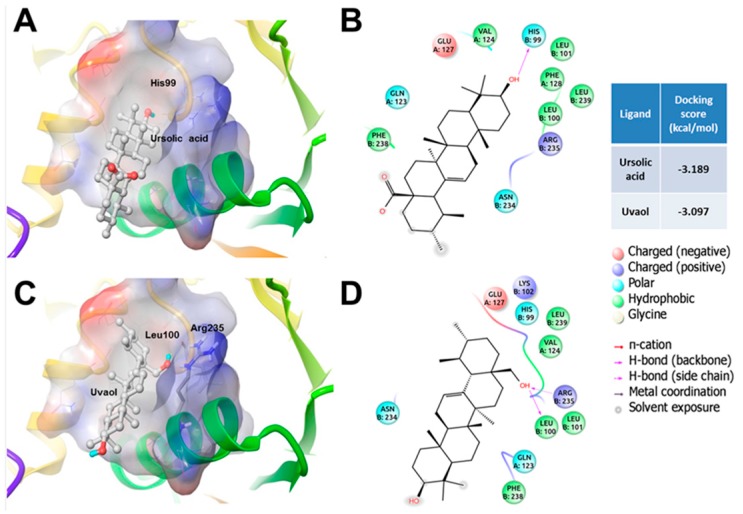
Binding mode of ursolic acid and uvaol into the CSE shared binding site: (**A**) short interactions (4.5 Å) of ursolic acid into the binding site; (**B**) 2D ligand interaction diagram of ursolic acid; (**C**) Short interactions (4.5 Å) of uvaol into the binding site; and (**D**) 2D ligand interaction diagram of uvaol.

As mentioned above, ursolic acid and uvaol showed preference for a binding site in CSE that shares residues from different chains, namely A and B. The proposed new binding site for the studied compounds is located *ca.* 12 Å from the cofactor pyridoxal-5′-phosphate (PLP) binding site. As well as in eNOS, the binding of the triterpenes to CSE could be stabilized by hydrogen bonds with polar residues, namely His99 for ursolic acid and Leu100 and Arg235 for uvaol in CSE, as well as by short contacts with other amino acids (Figure 10).

## 3. Discussion

In this work, we determined for the first time that the triterpenes ursolic acid and uvaol are the main non-polar vasodilator compounds in *P. serotina* fruits.

Ursolic acid and uvaol are ursane-type pentacyclic triterpenes widely distributed in different plant parts of medicinal and food species [28,29]. These compounds have been identified in several species of the genus *Prunus* [30,31,32,33,34], and particularly our working group isolated ursolic acid from the leaves of *P. serotina* [25]. According to the total weight obtained, the percentages of ursolic acid and uvaol are approximately 0.04% and 0.02%, respectively. Ursolic acid is recognized as a valuable medicinal natural product, which possesses several pharmacological activities, such as anticancer, antitumor, antiwrinkle [35,36]; antidiabetic [37]; anti inflammation [38], and anti-atherosclerotic [39], among others. On the other hand, uvaol, an important component of olive oil, has antiproliferative and antiatherogenic properties [40,41].

In previous studies, our research team and other groups reported that ursolic acid [25,42,43] and uvaol [44] produced vasodilation in rat aorta. In the present study, ursolic acid and uvaol relaxed the rat aorta by a mechanism that depends on the presence of the vascular endothelium. These results are consistent with an earlier survey, which demonstrated that ursolic acid causes an endothelium-dependent vasodilation [42]. In contrast, our findings regarding uvaol significantly disagree with a former investigation carried out by Rodríguez-Rodríguez *et al.* [44], who stated that uvaol elicited an endothelium-independent vasorelaxation, when tested in aortic rings from spontaneously hypertensive rats. In this regard, it has been described that some changes occur in vascular reactivity and morphology of thoracic aorta from normal and hypertensive rats [45,46,47]. The underlying etiology of these changes is multifactorial and strongly depends on the experimental model employed. Therefore, differences observed between our results and those reported by Rodríguez-Rodríguez *et al.* [44] might be attributed to either morphological or functional variations in the two experimental models.

The above-mentioned works by Aguirre-Crespo *et al.* [42] and Rodríguez-Rodríguez *et al.* [44] explored involvement of the NO/cGMP pathway in the vasodilatory effect of ursolic acid and uvaol, respectively. It was found that ursolic acid-induced vasorelaxation was blocked by l-NAME [42]. Contrastingly, relaxant response of uvaol was not modified by pretreatment with the same eNOS inhibitor [44]. In the present work, vasodilation by ursolic acid and uvaol was significantly reduced in the presence of l-NAME, which supports the hypothesis that these compounds activate the NO/cGMP pathway. Additionally, we investigated whether vasodilation elicited by these triterpenes involved participation of the H_2_S/K_ATP_ channel pathway. We found that, in fact, the CSE inhibitor, dl-propargylglycine, inhibited dilatation of rat aorta produced by ursolic acid and uvaol. One of the primary mechanisms by which H_2_S, synthetized in endothelial cells, induces vasorelaxation is activation of K_ATP_ channels located in vascular smooth muscle cells [48,49]. Therefore, participation of these latter channels in the vasodilation produced by both triterpenes was evaluated. Glibenclamide caused a significant reduction in the vasodilator effect of ursolic acid and uvaol, suggesting that K_ATP_ channels contribute to their effect. Taken together, these results provided additional evidence for the involvement of the H_2_S/K_ATP_ channel pathway in the triterpenes-induced vasorelaxation.

Moreover, simultaneous addition of submaximal concentrations of eNOS and CSE inhibitors almost completely blocked the vasorelaxant effect of either triterpenes. This potentiated response might be explained by a direct activation of both signaling pathways, which are closely interrelated and whose stimulation, according to numerous reports, triggers a complex cascade of interconnected chemical reactions that produce polysulfides and anionic *S/N*-hybrid species that are capable of producing vasodilation [50,51,52].

Further confirmation of our results was obtained when it was found that nitrite and H_2_S levels were significantly increased in aortic tissues, which had been incubated in the presence of either ursolic acid or uvaol. This evidence strongly suggests that both triterpenes activate the NO/cGMP and H_2_S/K_ATP_ channel pathways, likely through a direct activation of the enzymes eNOS and CSE.

An *in silico* analysis was performed to predict possible interactions between ursolic acid and uvaol and eNOS and CSE. The quaternary structure of eNOS consists of a homodimeric enzyme with several domains each monomer: a reductase-like domain, an oxidase-like catalytic site, a regulatory calmodulin binding loop, and four cysteines bound to Zn^2+^, which acts as a bridge between both monomers. Noteworthy, this enzyme needs five different cofactors to perform its function: FMN, FAD, NADPH, tetrahydrobiopterin and a heme group [53,54,55]. Thus, the great complexity of this protein has made difficult the crystallization of the whole enzyme, and up to now, there is no crystallographic structure featuring the aforementioned structural characteristics. Nevertheless, a number of crystallographic structures has been reported, which describe only the oxidase-like domain due to its importance for drug design and discovery of eNOS modulators [56].

In this work, a crystallographic structure of the oxidase-like domain was used with a molecule of tetrahydrobiopterin, a heme group and an eNOS inhibitor bound to the catalytic site. The catalytic site is located near the homodimeric interface, above the Zn-Cys cluster that stabilizes the homodimer and is conformed by several cavities: the substrate binding pocket above the heme group (S), two cavities that form the substrate entrance channel (C1 and C2), and a site that connects the aforementioned cavities (M) [43,57,58]. The binding sites of both triterpenes were located relatively far from the catalytic site, which indicated that the interaction of these compounds would not interfere with the binding of the substrate. We found that the docked triterpenes bind to specific amino acid residues that make up the C1 and C2 sites of eNOS, as described previously for ursolic acid and other triterpenes [43]. The docking scores of both triterpenes suggest that ursolic acid would have more affinity to the binding site than uvaol; thus, the effect on the quaternary structure of eNOS of the former would be greater than the effect of the latter. However, the difference of docking scores is less than 1 kcal/mol, thus the binding of both triterpenes could be considered to have a similar affinity for the binding site.

On the other hand, CSE is a homotetramer with a catalytic site dependent of pyridoxal-phosphate (PLP) bound to a lysine, and 3 interfaces: one, which is formed at the center of the tetramer and is stabilized by PLP, one, which divides the tetramer by half, and one, which is near the catalytic sites of each monomer. As opposed to eNOS, few crystallographic structures of CSE have been reported with the covalent inhibitor dl-propargylglycine (PAG), which revealed important residues that are in the catalytic site and are important for the proper function of this site [59]. The interface near the catalytic sites forms a “trench” with enough space to bind ligands and possibly constitutes a substrate access channel to the active site. As in the case of eNOS, the binding sites of both triterpenes were relatively far from the catalytic site and were stabilized by hydrogen bonds. Ursolic acid showed better docking score than uvaol; thus, the affinity and effect on the quaternary structure of CSE of the former would be greater than the affinity and effect of the latter. Also, the difference of docking scores is small; thus, a similar affinity to the binding site could be expected. Considering the influence of a small cofactor like PLP in the stabilization of the quaternary structure of CSE, the binding of these triterpenes to the interface could have a great impact in the structure of the catalytic site.

The results derived from the *in silico* study support the hypothesis that ursolic acid and uvaol might directly activate eNOS and CSE. Here, we show for the first time that ursolic acid and uvaol are able to bind with high affinity to an allosteric binding site in both enzymes. Once the triterpenes bind to eNOS and CSE, the proposed binding site found in this work could act as an allosteric site or could stabilize the quaternary structure of the active site, resulting in the activation of these enzymes. Noteworthy, with these computational studies, a promissory new binding site that could act as an allosteric control site has been found for both enzymes. These calculations agree with the experimental observations, making possible to suggest a mode of action for the triterpenes. In forthcoming works, more calculations will be performed to corroborate these findings and to assess precisely the main interaction residues and the nature of those interactions.

The results obtained from the *ex vivo*, *in vitro*, and *in silico* studies carried out in the present research constitute a solid starting point for future investigations designed to further confirm direct activation of eNOS and CSE by these ursane type triterpenes in the search of prototypes for the development of novel antihypertensive drugs.

## 4. Materials and Methods

### 4.1. Plant Material and Reagents

The mature fruits of *P. serotina* were collected in orchards located in Huejotzingo, Puebla, México. The specimens were identified (*P. serotina* voucher 1414006) and deposited in the Herbario Nacional de México, Instituto de Biología, Universidad Nacional Autónoma de México.

The reagents and solvents used in the bio-directed chemical study were obtained from JT Baker (Phillipsburg, NJ, USA). The standards for the pharmacological and biochemical assays were purchased from Sigma-Aldrich (St. Louis, MO, USA).

### 4.2. Experimental Animals

Wistar male rats (250–300 g) were used for the pharmacological study; they were provided by the Institute of Neurobiology of the National Autonomous University of Mexico, Campus Juriquilla. All experiments were performed according to the indications of the Mexican Official Standard NOM-062-ZOO-1999 concerning the production, care and use of laboratory animals (Norma Oficial Mexicana, 2001).

### 4.3. Collection and Preservation of Plant Material

The harvested fruits were transported to the Laboratory of Natural Products of the Chemistry Faculty of the Autonomous University of Queretaro, in Queretaro, Mexico. Subsequently, the harvested fruits were selected based on their physical integrity, washed with water and dried completely. The fruits were segmented and, after the removal of seeds, frozen at −70 °C, and lyophilized.

### 4.4. Bio-Directed Chemical Study of the Dichloromethane Extract Obtained from the Fruits of P. serotina

#### 4.4.1. Preparation of the Dichloromethane Extract from the Fruits of *P. serotina*

To obtain the extract, the lyophilized fruits were subjected to maceration with dichloromethane for one week in a 1:3 ratio (*w*/*v*). At the end of this time, the extract was filtered and the solvent was removed using a rotary evaporator. The resulting extract was stored at −20 °C.

#### 4.4.2. Bio-Directed Fractionation of the Dichloromethane Extract and Purification of Ursolic Acid and Uvaol

The dichloromethane extract was subjected to an initial fractionation by column chromatography on normal phase using an open silica gel column (100 g, Kiesegel 60 Merck 70–230 mesh, particle size of 0.063–0.200 mm). Hexane and acetone were used as eluents in various proportions to obtain the fractions, which were grouped according to their chromatographic similarity.

The resulting fractions were subjected to pharmacological evaluation using the isolated rat aorta assay to identify the bioactive fractions. The activity of each fraction was evaluated at a concentration equal to the mean effective concentration (EC_50_) of the dichloromethane extract (90 µg/mL).

The purification of ursolic acid and uvaol from the active fraction PD-III was performed in the same manner as the initial fractionation: by open column chromatography on silica gel, using hexane and acetone in different proportions as eluents. The purity of ursolic acid and uvaol was verified by thin-layer chromatography and by determining their melting points.

#### 4.4.3. Determination of the Chemical Structure of Ursolic Acid and Uvaol

The structural elucidation of ursolic acid and uvaol was carried out by the analysis of their proton nuclear magnetic resonance (^1^H-NMR) and carbon-13 (^13^C-NMR) spectra. These compounds were identified by direct comparison with an authentic sample and by comparing their spectroscopic constants with those reported in the literature [60,61].

### 4.5. Determination of the Vasodilator Effect and Elucidation of the Mechanism of Action of Ursolic Acid and Uvaol

#### 4.5.1. Isolated Rat Aorta Assay

Rats were sacrificed by decapitation. The descending thoracic aorta was removed and placed in cold Krebs-Henseleit solution (126.8 mM NaCl, 5.9 mM KCl, 2.5 mM CaCl_2_, 1.2 mM MgSO_4_, 1.2 mM KH_2_PO_4_, 30 mM NaHCO_3_ and 5 mM D-glucose; pH 7.4). The aorta was cut into rings of 4 to 5 mm, which were mounted in incubation chambers with 7 mL of Krebs-Henseleit solution at 37 °C and constant bubbling with a mixture of 95% O_2_ and 5% CO_2_. The tissues were subjected to a basal tension of 1.5 g and were allowed to stabilize for 60 min. Once the tissues reached equilibrium, the segments were pre-contracted with a 100 mM KCl solution for 15 min to stimulate arterial smooth muscle. Subsequently, KCl was removed, allowing the aorta to stabilize until it reached basal tension again. After stabilization of the tissue, the test compounds were evaluated in a concentration range of 1 to 1000 µg/mL on the aortic rings pre-contracted with phenylephrine (1 µM). The endothelial integrity of the preparations was determined by adding acetylcholine (1 μM) to the chamber bath. Only arteries with a vasodilator response more than 70% were considered endothelium-intact. The changes in tension caused by the tested concentrations were detected by Grass FT03 force transducers coupled to a Grass 7D Polygraph; they were expressed as percentages of relaxation based on the contraction generated by adding phenylephrine [62,63].

#### 4.5.2. Participation of the Endothelium in the Vasodilator Response

To determine whether the vasodilator response was dependent on the vascular endothelium, assays on aorta segments without endothelium were performed. In these experiments, the endothelium was mechanically removed by gently rubbing the internal aortic ring surface with a wood rod. The absence of endothelium was confirmed at the start of the experiment, showing that the addition of 1 µM of acetylcholine (ACh) did not induce more than 5% relaxation.

#### 4.5.3. Evaluation of the Participation of the NO/cGMP and the H_2_S/K_ATP_ Channel Pathways in the Vasodilator Response

Involvement of these biochemical pathways in the vasodilator effect of the test compounds was evaluated by incubating the tissue for 20 min in the presence of inhibitors of specific enzymes of each of these pathways: (1) NO/cGMP pathway: 100 µM NG-nitro-l-arginine methyl ester (l-NAME, inhibitor of NO synthase, NOS); and (2) H_2_S/K_ATP_ channel pathway: 10 mM dl-propargylglycine (PAG, inhibitor of cystathionine-γ-lyase, CSE). To determine the involvement of the activation of the K_ATP_ channels in the vasodilator effect of the test compounds, aortic segments were incubated for 20 min in the presence of 1 µM glibenclamide, a blocker of these channels, before adding the test compound [64,65].

Simultaneous inhibition of both enzymes, eNOS and CSE, was assessed employing submaximal concentrations of their respective inhibitors: 1 µM l-NAME and 1 mM PAG.

### 4.6. Increase in NO and H_2_S Levels Elicited by Ursolic Acid and Uvaol

#### 4.6.1. NOS Enzymatic Activity Assay

Aortic rings, previously incubated for 30 min with ursolic acid (EC_50_ = 21.5 ± 3.5 μg/mL final concentration) or uvaol (EC_50_ = 19.3 ± 2.5 μg/mL final concentration) or ACh (1 × 10^−4^ M final concentration, positive control), were homogenized in phosphate buffer solution (PBS) 100 mM, pH 7.4. Homogenates were centrifuged at 24,400× *g* for 20 min and the supernatant solutions were filtered. One hundred millimeter triplicate samples of filtered solutions were incubated for 3 h at room temperature in 96-well plates with PBS (pH 7.4) containing nitrate reductase (50 mU/100 µL of sample) and NADPH (80 µM, final concentration). After the incubation time, 50 µL of 1% sulfanilic acid and 50 µL of 0.2% *N*-naphthyl ethylenediamine were added to induce formation of a colored azo compound. Absorbance was measured at 540 nm and samples concentrations were calculated with a linear equation based on a NO_2_^−^ calibration curve [66,67].

#### 4.6.2. CSE Enzymatic Activity Assay

Aortic segments were homogenized in PBS pH 7.4 with a protease inhibitor (Sigmafast protease inhibitor cocktail tablets, EDTA free) and centrifuged at 16,945× *g* for 15 min at 4 °C. The supernatant (350 µL) was added with: 50 µL of the triterpenes at the EC_50_ obtained from the concentration-response curves of ursolic acid (21.5 ± 3.5 μg/mL final concentration) and uvaol (19.3 ± 2.5 μg/mL final concentration) or ACh (1 × 10^−4^ M final concentration), 25 μL of 2 mM pyridoxal 5′phosphate, and 25 μL of 10 mM l-cysteine. This mixture was left to react for 60 min in a 37 °C shaking water bath and the reaction was ended by adding 250 μL of 1% zinc acetate to trap H_2_S. Afterwards, 250 μL of 10% trichloroacetic acid and 200 μL of 20 mM *N*,*N*-diethyl-*p*-phenylenediamine sulphate in 1.2 M HCl were added, followed by addition of 200 μL of 30 mM iron trichloride in 1.2 M HCl. After 20 min, absorbance was measured at 670 nm and H_2_S concentration was calculated against a calibration curve of standard NaHS solution (5 to 100 µM) [68,69].

### 4.7. In silico Studies

*In silico* studies were conducted by using Schrödinger Maestro Suite 2014-1 (Schrödinger, LLC, New York, NY, USA, 2014). File preparation and docking studies were performed on this software.

#### 4.7.1. Protein Preparation

The crystallographic structures of eNOS (PDB: 1D0C) and CSE (PDB: 3COG) were suitably obtained and prepared using Protein Preparation Wizard [70] available in Maestro (Protein Preparation Wizard; Epik version 2.7, Schrödinger, LLC, 2013; Impact version 6.2, Schrödinger, LLC, 2014; Prime version 3.5, Schrödinger, LLC, 2014). Briefly, all missing residues were added, all ligands except cofactors and crystallographic waters were removed. Finally, the structures were protonated to physiological pH (7.0 ± 0.5) and the protonated structures were minimized using Optimized Potentials for Liquid Simulations (OPLS) force field.

#### 4.7.2. Ligand Preparation

The structures of ursolic acid and uvaol were obtained from PubChem [71] (entries 64945 and 92802, respectively) and prepared with LigPrep on Maestro (LigPrep, version 2.9, Schrödinger, LLC, 2014), selecting the minimized structure for docking studies.

#### 4.7.3. Docking Studies

These studies were conducted by using Glide 6.2 [72,73,74] with default parameters, available in Maestro (Glide, version 6.2, Schrödinger, LLC, 2014). First, a blind docking protocol was used to assess the highest affinity site for the compounds; once the binding site was located, docking calculations were performed using Glide 6.2 Extra Precision (XP) in the neighborhood of the binding site using the default parameters. As a validation of the protocol used, redocking of crystallographic ligands were made on eNOS.

### 4.8. Statistical Analysis

Six evaluations were performed for each of the concentrations of the test substances. The results are expressed as the mean ± standard error of the mean (SEM). The experimental data generated during each evaluation were fitted to a sigmoidal equation and plotted using the software PRISM 5.0 GraphPad (Graph Pad Software Inc., San Diego, CA, USA) to obtain concentration-response curves. The EC_50_ was calculated for each case.

Concentration-response curves were analyzed by repeated-measures two-way ANOVA, using the statistical program PRISM 5.0 GraphPad, followed by the Bonferroni test to evaluate any significant differences between the means. In Figure 3, comparison was made between the curves of each triterpene in the presence and absence of endothelium. In Figure 4, Figure 5 and Figure 6, concentration–response curves of ursolic acid and uvaol were compared with the respective concentration–response curves obtained in the presence of the different inhibitors tested. The concentration–response curves of ursolic acid and uvaol were only constructed once (Figure 3), and they were used as controls in Figure 4, Figure 5 and Figure 6.

Comparisons between two populations in the NOS and CSE enzymatic activity assays were made using a one-way ANOVA, followed by a Tukey’s test.

## 5. Conclusions

In this paper, we report for the first time that the main non-polar vasodilatory compounds in *P. serotina* fruits are ursolic acid and uvaol. In addition, this study is the first that proposes involvement of the H_2_S/K_ATP_ channel pathway in the vasodilator effect induced by these triterpenes. Participation of the NO/GMPc pathway in the vasodilation produced by these compounds was also confirmed. Moreover, we demonstrated that ursolic acid and uvaol are able to activate simultaneously both NO/GMPc and the H_2_S/K_ATP_ channel pathways, which results in a synergistic vasorelaxant effect. *In silico* studies indicated, that these compounds might activate both eNOS and CSE by binding to an allosteric control site located in the enzymes. Therefore, both secondary metabolites can be considered as prototype molecules with a novel mechanism of action for the development of drugs useful in the prevention and/or treatment of cardiovascular diseases, such as hypertension.
